# Health systems performance in sub-Saharan Africa: governance, outcome and equity

**DOI:** 10.1186/1471-2458-11-237

**Published:** 2011-04-16

**Authors:** Anna E Olafsdottir, Daniel D Reidpath, Subhash Pokhrel, Pascale Allotey

**Affiliations:** 1 Centre for Public Health Research, Brunel University, West London, UK; 2 Global Public Health, Jeffrey Cheah School of Medicine and Health Sciences, Monash University, Bandar Sunway, Malaysia

## Abstract

**Background:**

The literature on health systems focuses largely on the performance of healthcare systems operationalised around indicators such as hospital beds, maternity care and immunisation coverage. A broader definition of health systems however, needs to include the wider determinants of health including, possibly, governance and its relationship to health and health equity. The aim of this study was to examine the relationship between health systems outcomes and equity, and governance as a part of a process to extend the range of indicators used to assess health systems performance.

**Methods:**

Using cross sectional data from 46 countries in the African region of the World Health Organization, an ecological analysis was conducted to examine the relationship between governance and health systems performance. The data were analysed using multiple linear regression and a standard progressive modelling procedure. The under-five mortality rate (U5MR) was used as the health outcome measure and the ratio of U5MR in the wealthiest and poorest quintiles was used as the measure of health equity. Governance was measured using two contextually relevant indices developed by the Mo Ibrahim Foundation.

**Results:**

Governance was strongly associated with U5MR and moderately associated with the U5MR quintile ratio. After controlling for possible confounding by healthcare, finance, education, and water and sanitation, governance remained significantly associated with U5MR. Governance was not, however, significantly associated with equity in U5MR outcomes.

**Conclusion:**

This study suggests that the quality of governance may be an important structural determinant of health systems performance, and could be an indicator to be monitored. The association suggests there might be a causal relationship. However, the cross-sectional design, the level of missing data, and the small sample size, forces tentative conclusions. Further research will be needed to assess the causal relationship, and its generalizability beyond U5MR as a health outcome measure, as well as the geographical generalizability of the results.

## Background

In the World Health Report 2000, a health system is discussed in terms of "all the organizations, institutions and resources that are devoted to producing health actions" [1] (p xi). Notwithstanding this very broad description of a health system, when it comes to the analysis of health systems performance, the operational (nonprocess) approaches have tended to be narrow, focusing on those aspects of the system that relate directly to the delivery of healthcare. This is particularly apparent in the analyses of health systems performance in high income countries [2-5]; and does not appear to have been materially influenced by the development of wider frameworks of analysis [6].

In high income countries the lack of distinction between a health system and a healthcare system may be appropriate. With few exceptions, the OECD countries and the high income non-OECD countries have stable governments and well developed national infrastructure, including functioning commercial and financial systems, embedded utility grids delivering clean water and energy; systems that facilitate communication and transportation; liveable national housing; a functioning judicial and educational system; etc. In these settings, population health gains are part of a marginal game often based on incremental improvements to an existing healthcare system that operates within the established context of high quality national infrastructure [7,8]. It is surprising, therefore, that the analysis of health systems performance in low income countries is also based largely on an analysis of systems that deliver care, despite the absence of the wider infrastructure required to support functioning healthcare systems [9-12].

Some would explain the focus by arguing the inappropriateness of looking at non-healthcare factors, because an analysis of non-healthcare factors effectively holds the health sector to ransom - making it accountable for those determinants of health that do not fall within its direct control [13]. The difficulty with this position is the overwhelming body of evidence that demonstrates the critical role of socio-economic, environmental, and other structural determinants of health [14]. Furthermore, ignoring broader structural factors assumes that one can "strengthen" a health system without regard to the economic, social, and physical context within which the delivery of healthcare is supposed to occur. If the health system is not held accountable for these larger determinants, argued Murray and Frenk, there will be no advocate in a country for addressing them (p.727) [13]. If not us then who?

For high income countries the question of the appropriateness of using the healthcare system as a proxy for a health system more broadly is moot [8]. In low income countries, however, with poor infrastructure, often weak political, commercial, financial and regulatory systems, to exclude non-healthcare system artefacts from the analysis of health systems performance relies on a much more tenuous foundation. It is, thus, unlikely that health systems performance in low income countries can be reduced to an analysis of the incremental health gains associated with improvements to the healthcare system. This point is clearly demonstrated again by ongoing challenges to universal access to healthcare [15].

In this article, guided by the broader definition of health systems provided by the World Health Organization, we attempt to refocus the analysis of health systems performance on one particular non-healthcare related issue. Specifically, we are interested in the relationship between governance and the overall performance of the health system. Other structural factors such as the finance and the economy [16], the education system [17,18], and physical infrastructure [19], have all been shown to be related to health systems performance. Governance, however, has emerged relatively recently as a measurable, structural artefact of countries that could be related to performance in a number of domains [20,21], including health [22]; but there is relatively little research that has looked at this.

## Methods

An ecological analysis of cross sectional data was performed to look at the association between governance and health systems performance in the countries of the WHO African region (AFRO), independent of other known healthcare and non healthcare artefacts. The AFRO region was chosen because of the concentration of low income countries and the low probability that many of the countries will achieve the 2015 MDG targets [23]. The analysis was carried out using the most up-to-date, publicly available country-level data from the Statistical Information System of the World Health Organization (WHOSIS) [24] and the Mo Ibrahim Foundation (MIF) [25].

### Measures

#### Outcomes

In keeping with the approach articulated in the World Health Report 2000 (p. xi) [1] health systems performance was operationalised in terms of the two separate dimensions: health outcome (i.e., death and morbidity) and health equity (i.e., the fairness of the distribution of health outcomes). The specific measure chosen for health outcome was a country's under five mortality rate (U5MR); i.e., the probability of a child's death before reaching the age of five. Although U5MR is a narrow operational definition of a population's health outcome, measures of child health such as the U5MR and the Infant Mortality Rate have been successfully used as general indicators of population health because they are sensitive to both structural changes and to rising epidemics that affect the wider population [26-29]. Data on U5MR were available for all 46 countries in the AFRO region.

A fair or equitable health system would be one that produces equivalent health outcomes for the rich and the poor [30,31]. Using the U5MR as a direct measure of health outcome, health equity was operationalised as the ratio of the U5MR in the wealthiest quintile to that of the U5MR in the poorest quintile. Data on the quintile ratio of the U5MR were available for 30 of the 46 countries in the AFRO region. Combining the two dimensions of health outcome and equity, a health system that was performing well would have a low U5MR and a U5MR quintile ratio approaching 1.

#### Governance

Governance was defined here as "a process whereby societies or organizations make their important decisions, determine whom they involve in the process and how they render account" (p1) [32]. Governance can be thought of as a structural artefact of a society; notwithstanding the fact that it is defined in terms of process. This is because governance occurs within social structures created for the purposes of facilitating the process. Two AFRO-relevant sources of governance measures are available: the World Bank governance index [20] and the Ibrahim Index of African Governance [25]. These indices are based on data drawn from a diversity of international organisations, non-governmental organisations, survey institutes and think tanks. The Ibrahim Index was selected because of its greater contextual relevance to the AFRO region. It was developed in cooperation with the Harvard Kennedy School and it aggregates third party data (inter alia from the World Bank) and original data to create an index that ranks countries in Africa according to governance quality. The index assesses nations against 57 different measures. As the total index included child mortality two sub-indicators from the 2006 Ibrahim Index that did not include child mortality in the measure were used to operationalise governance: (i) "Rule of Law, Transparency and Corruption" (RLTC), and (ii) "Sustainable Economic Opportunity" (SEO)^i) ^[25]. RLTC captures governance matters such as the ratification of legal norms, judicial independence, and public sector corruption. SEO captures matters such as wealth creation, macroeconomic stability, and the "arteries of commerce" including the extent of the sealed road network, and the availability of electricity.

The Index as a whole, and the sub-scale were recently, independently analysed and found to be both reliable (i.e., the scales could be independently reproduced) and valid (i.e., they captured, and reflected the position of the countries on the specific "pillars" of governance) [33].

#### Covariates

Four additional, potential confounding factors were included in the analysis reflecting healthcare, financing/economy, education and physical infrastructure. For each factor a number of indicators were used. The choice of indicators was driven in part by pragmatics, including the availability of the data, and concordance with the underlying factor.

For healthcare there were three indicators: (i) the percentage of births attended by skilled health personnel, (ii) the percentage of one year olds immunised with three doses of diphtheria, tetanus toxoid and pertussis (DTP3), and (iii) the number of hospital beds per 10,000 populations. The first two of these indicators were most directly relevant to child health, but could broadly be regarded as indicators of the capacity of the wider healthcare system. For financing/economy two indicators were considered: (i) per capita total expenditure on health, and (ii) gross national income (GNI) per capita. Both financing indicators were measured in international dollars adjusted for purchasing power parity (ppp int.$). For education three indicators were considered: (i) adult literacy, (ii) the net primary school enrolment ratio male, and (iii) the net primary school enrolment ratio female. Net primary school enrolment ratio is the ratio of the number of children of official school age enrolled in school to the number of children of official school age in the population. The net primary school enrolment ratio male was ultimately excluded because it was highly correlated with the net primary school enrolment ratio female (r = .95). For physical infrastructure two indicators were included: (i) the percentage of the population with sustainable access to safe drinking water sources, and (ii) the percentage of the population with sustainable access to adequate sanitation.

### Analysis

The analysis of the relationship between U5MR and the U5MR quintile ratio, and governance and the other covariates was conducted using multiple linear regression. The analysis was complicated by issues of confounding, associated with moderate to high inter-correlations between the covariates and governance, and missing data. This is discussed in more detail in the Results and related issues are described in the following.

The U5MR data were available for all the 46 countries in the AFRO region but the quintile ratio data was only available for 30 out of the 46 countries. The final sample size in the multivariate analyses was affected by missing data in the covariates.

A small sample size relative to the number of independent variables or covariates in a multivariate analysis can result in (a) over fitting, (b) unstable estimated coefficients, and (c) in the case of this analysis, an increased likelihood of a Type II error. A Type II error arises when one fails to reject the null hypothesis when it is actually false. A small sample size and many covariates reduce the likelihood of identifying a significant relationship between the health systems performance measures and the governance measures, even if such a relationship truly exists. Thus, the worst case scenario is that the analysis described is conservative, and fails to identify a relationship where one actually exists. This does not affect the risk of a Type I error, of falsely rejecting the null hypothesis.

Three linear models were fitted separately for U5MR and the U5MR quintile ratio. The first model measured the strength of the unadjusted bivariate association between the health systems performance, governance and the covariates reflecting healthcare, finance, education, and physical infrastructure. The second model measured the strength of the adjusted association between the health systems performance and the covariates in the absence of the measures of governance. The third model measured the strength of the association between the health systems performance and governance after adjusting for the covariates reflecting healthcare, finance, education, and physical infrastructure. The estimated standardised coefficients (β's) from the regression analysis are reported, because they simplify the comparison of the relative contribution of the independent variables and covariates. A sensitivity analysis was conducted to look at, among other things, the change in the estimates when covariates that reduced the sample size (due to missing data) were removed, and the relationship between U5MR and the U5MR quintile ratio with missing data in the covariates.

Prior to any formal analyses, GNI per capita was log-transformed to enable a better fit to the outcome measures.

### Missing Data

Missing was an issue for U5MR and the quintile-ratio data because of the small size of the data set, and the percentage of the countries that did not have complete data for all covariates. Multiple imputation of missing data was attempted, but the data models failed to converge [34].

An examination of the relationship between U5MR and missing covariates, for countries with and without missing data, showed no significant relationship. Similar checks were made for the quintile ratio. Missing data, nonetheless, remain a concern and this is discussed further.

## Results

An overview of the governance factor (two indicators), and the four covariate factors (a total of 9 indicators) as well as descriptive statistics for U5MR and the U5MR quintile ratio are shown in Table 1.

**Table 1:** Descriptive statistics for U5MR, U5MR quintile ratio and indicators used in the analysis.

*Health Systems Performance*	n	Mean	SD	Min	Max
U5MR^1^	46	138.5	62.5	13	269
U5MR quintile ratio	30	1.8	0.66	0.9	4

*Healthcare*					
Births Attend %	45	55.5	22.2	6	99
DTP3^2 ^Immunisation %	46	77.5	18.7	20	99
Hospital Beds/10,000	44	13.7	11.2	1	57

*Finance*					
GNI^3 ^(untransformed) $	45	2,923	3,995	260	16,620
Health Expenditure $	46	153	200	15	869

*Education*					
Enrollments (female) %	45	69.7	20	36	100
Enrollments (male) %	45	74.1	15.8	40	99
Adult Literacy %	42	62.2	20.6	23.6	91.8

*Physical Infrastructure*					
Water %	46	67.7	16.2	42	100
Sanitation %	45	34.5	19.7	5	94

*Governance*					
RLTC^4^	46	54.2	14.4	24.3	86.1
SEO^5^	46	41.8	10.9	23.3	71.4

^1 ^U5MR: Under five mortality rate

^2 ^DTP3: Three doses of diphtheria, tetanus toxoid and pertussis

^3 ^GNI: Gross National Income

^4 ^RLTC: Rule of Law, Transparency and Corruption

^5 ^SEO: Sustainable Economic Opportunity

The U5MR ranged between 13 and 269 per 1,000 live births; and the quintile ratio ranged between 0.9 (slightly better outcomes for the poorest than the wealthiest quintile) and 4 (much worse outcomes for the poorest quintile). The countries wealth and therefore the ability to spend on health differed vastly. GNI per capita ranged from USD$260 to USD$16,620 and health expenditure from USD$15 to USD$869.

As a preliminary step, the relationship between the two selected dimensions of health systems performance (U5MR and the U5MR quintile ratio) was examined visually and using exploratory statistics. There was no discernible relationship between the two health systems performance measures and the correlation was low (r = -.15; 95% CI: -.49-.22). This reinforced the notion of separate outcome and equity dimensions in health systems performance.

### Health Outcome (U5MR)

The U5MR data was modelled first (Table 2). Model 1 shows the bivariate relationship between, U5MR, the 9 covariates, and the two measures of governance. In a bivariate analysis, the parameter estimate (β coefficient) is the same as Pearson's product moment correlation (r). All 9 covariates, and the measures of governance were significantly associated with U5MR. The two governance measures had the strongest bivariate associations with U5MR (~.7), followed by sustainable access to safe drinking water, the percentage of female children enrolled in school, and the number of hospital beds per 10,000.

**Table 2:** Three models estimating the relationship between health outcome (U5MR), governance (RLTC and SEO) and 9 covariates representing healthcare, finance/economy, education, and physical infrastructure^**1**^.

	U5MR					
	
	Model 1 (41 < n <45)		Model 2 (n = 37)		Model 3 (n = 37)	
	β/r	p	β	p	β	p
*Healthcare*						
Births Attended %	-.56	<.001	-.27	.20	-.31	.06
Immunisation %	-.44	<.001	.00	.98	.01	.93
Hospital Beds/10,000	-.63	<.001	.03	.90	.02	.90
*Finance*						
GNI^2 ^(transformed) $	-.47	<.001	.09	.68	.41	.04
Health Expenditure $	-.50	<.001	.03	.87	.18	.27
*Education*						
Enrolments (female) %	-.64	<.001	-.57	<.001	-.32	.02
Adult Literacy %	-.55	<.001	.03	.86	.20	.19
*Physical Infrastructure*						
Water %	-.67	<.001	-.41	.03	-.10	.52
Sanitation %	-.35	.02	.10	.47	.15	.20
*Governance*						
RLTC^3^	-.70	<.001			-.12	.42
SEO^4^	-.72	<.001			-.90	<.001

Adjusted R-squared			.59		.76	

^1^Model 1 examines the bivariate relationship between, U5MR, and the 9 covariates, and the two measures of governance. Model 2 examines the multivariate model of the linear relationship between U5MR and the covariates in the absence of the measures of governance. Model 3 replicated Model 2, with the addition of the two governance measures.

^2^GNI: Gross National Income

^3^RLTC: Rule of Law, Transparency and Corruption

^4^SEO: Sustainable Economic Opportunity

Model 2 represents the multivariate model of the linear relationship between U5MR and the covariates in the absence of the measures of governance. This model was included so that a comparison could be made between it and a fuller model including the measures of governance. This would help to illuminate the strength of the relationship between U5MR and governance (i.e., when Model 2 and Model 3) are compared. Only 37 of the 46 countries which had U5MR data were available for this analysis, because of missing data in the covariates. Model 2 accounted for around 60% of the available variance (Adjusted R^2 ^= .59). After adjustment, only two of the covariates retained a significant association with U5MR, sustainable access to safe drinking water (β = -.41, p = .03) and the percentage of female children enrolled in school (β = -.57, p < .001). The negative coefficients indicated that improvements in the availability of water, and improvements in the enrolment of female children in school, were significantly associated with reductions in U5MR. These two significant associations were in the expected direction.

Model 3 replicated Model 2, with the addition of the two governance measures (n = 37). The model accounted for around 16% more of the available variance (Adjusted R^2 ^= .76), and identified a significant association between one of the governance indicators (sustainable economic opportunities; β = -.90, p < .001) and U5MR, after adjustment for the other governance indicator (rule of law, transparency and corruption; β = -.12, p = .42) and the other covariates. The governance indicator for sustainable economic opportunities (SEO) had the largest standardised coefficient of any of the variables included in the analysis. Female enrolments remained significant (β = -.32, p = .02), while sustainable access to safe drinking water was no longer significant in the fully adjusted model (β = -.10, p = .52). GNI per capita, however, became significant in the fully adjusted model (β = .41, p = .04) and showed, counter intuitively, that increases in GNI per capita were associated with increases in U5MR. The kind of instability in parameters seen here is a common issue where there are large numbers of covariates in relation to the sample size, and necessarily raised concerns about the stability of the estimated relationship between governance and U5MR.

A sensitivity analysis was conducted to examine the stability of the estimated association between the governance indicators and U5MR. In essence, by varying combinations of covariates, it was possible to increase the sample size, and examine the stability of the association between SEO and U5MR. The direction and broad magnitude of the association between governance and U5MR did not change with the modelling strategy. For example, removing adult literacy as a covariate increased the available sample size from 37 to 40 countries, resulting in a poorer fit of the model with the data (adjusted R^2 ^= .64), but SEO remained significantly associated with U5MR (β = -.76, p < .001). Furthermore, because the SEO indicator included a measure of wealth, a further sensitivity analysis was conducted using an adjusted SEO measure, having removed the effect of GNI per capita. The results remained materially the same.

### Health equity (U5MR quintile ratio)

The pattern of results was distinctly different for the models of the U5MR quintile ratio data (Table 3). Model 4 shows the bivariate relationship between, the 9 covariates, the two measures of governance, and the U5MR equity ratio. No indicators of healthcare, education, or physical infrastructure were significantly correlated with the U5MR quintile ratio data (p < .05). SEO was significantly correlated with the U5MR equity ratio (β = .42, p = .02), as was health expenditure (β = .53, p < .001). A number of the indicators had p-values less than .1, including births attended (healthcare) (β = .32, p = .09), GNI per capita (finance) (β = .35, p = .06), sanitation (physical infrastructure) (β = .33, p = .08), and RLTC (governance) (β = .32, p = .09).

**Table 3:** Three models estimating the relationship between health equity (U5MR quintile ratio), governance (RLTC and SEO) and 9 covariates representing healthcare, finance/economy, education, and physical infrastructure^1^.

	U5MR Quintile Ratio					
	
	Model 4 (28 < n <30)		Model 5 (n = 25)		Model 6 (n = 25)	
	β/r	p	β	p	β	p
*Healthcare*						
Births Attended %	.32	.09	.31	.30	.32	.34
Immunisation %	.24	.19	.15	.55	.10	.72
Hospital Beds/10,000	.07	.73	-.73	.04	-.73	.06
*Finance*						
GNI (transformed) $	.35	.06	-.04	.92	-.14	.77
Health Expenditure $	.53	<.001	.88	.01	.85	.02
*Education*						
Enrolments (female) %	.25	.20	-.18	.44	-.24	.40
Adult Literacy %	.23	.24	.42	.18	.36	.35
*Physical Infrastructure*						
Water %	.15	.43	-.42	.21	-.47	.25
Sanitation %	.33	.08	.27	.23	.27	.25
*Governance*						
RLTC	.32	.09			.10	.77
SEO	.42	.02			.19	.71

Adjusted R-squared			.30		.21	

^1^Model 4 examines the bivariate relationship. Model 5 examines the multivariate relationship between the covariates and health outcome in the absence of the measures of governance. Model 6 examines the multivariate relationship between health outcome and governance, adjusting for the covariates.

Model 5 represents the multivariate model of the linear relationship between the U5MR quintile ratio and the covariates in the absence of the measures of governance (n = 25); and it accounted for 30% of the variance (Adjusted R^2 ^= .30). Only two of the covariates were, independently, significantly associated with the U5MR quintile ratio. The first of these was the availability of hospital beds per 10,000 (β = -.73, p = .04). As the number of hospital beds per 10,000 increased, so there was an associated improvement in health equity. The second independently significant covariate associated with the U5MR equity ratio was per capita total expenditure on health (β = .88, p = .01). Increases in per capita health spending were associated with worsening equity.

Model 6 replicated Model 5, with the addition of the two governance measures (n = 25). The addition of the governance indicators resulted in a poorer fit to the data (Adjusted R^2 ^= .21), and neither SEO (sustainable economic opportunities; β = .19, p = .71) nor RLTC (rule of law, transparency and corruption; β = .10, p = .77) were significantly associated with the U5MR quintile ratio. In the fully adjusted model, the only covariate that remained significantly associated with the U5MR quintile ratio was per capita total expenditure on health (β = .85, p = .02).

## Discussion

The primary interest was in the association between health systems performance (as measured by a health outcome -U5MR - and a health equity measure - the U5MR quintile ratio) and a non-healthcare factor, namely governance. There was no apparent relationship between the health outcomes dimension of health systems performance (U5MR) and the health equity dimension (U5MR quintile ratio). This is noteworthy for two reasons. First, and purely from an analytic perspective, a weak relationship reinforces the need to model the two dimensions of health systems performance independently. Second, and with a policy perspective in mind, in the absence of a strong relationship between the dimensions there is a need to take both seriously, and not assume that addressing one will necessarily address the other.

The analysis showed that governance, in particular "sustainable economic opportunities," was significantly associated with health outcome measured as under five mortality rate and remained so even after controlling for the other healthcare and non-healthcare factors (see also [22]). Furthermore, the result was independent of the approach taken to the analysis of the data. Although a causal relationship cannot be established in an ecological, cross sectional analysis of this kind, the results leave open the possibility that good governance helps to create an environment in which a health system can perform well, and certainly invites further research to examine the matter of causation.

In sharp contrast, and notwithstanding a significant bivariate association between governance (SEO) and health equity, there was no significant association in the multivariate analysis. Given the weak relationship between health outcomes and health equity, this may not be entirely surprising. It raises, questions, however, about which non-healthcare factors should be future targets for investigation, and no clear answer presents itself.

The results should be regarded as preliminary, and limited by the amount and quality and type of data. The number of countries contributing data was small relative to the number of variables included in the analyses, and the situation was worse for the analysis of equity than it was for the analysis of health outcomes. Confounding was an issue. The independent measures are intercorrelated and correlated with the outcome measures. Negative confounding is the likely explanation, for instance, of the anomalous relationship in which increased country GNI was associated with a worse U5MR after the inclusion of governance (Table 2: Model 3). GNI and SEO are both negatively associated with U5MR (SEO much stronger) at the same time as GNI and SEO are highly positively related which might cause the GNI to turn falsely positive when run in a multivariate regressions including SEO. Longitudinal data (allowing for the inclusion of time-lags) would strengthen the analysis considerably. However, the dangers of the ecological fallacy are inescapable. Issues of endogeneity will also haunt any attempt to jump from an analysis of association to a causal inference. Causal order, lurking variables, and a lack of appropriate instrumental variables are all impediments, compounded by issues of data quality and the necessarily limited sample of countries. Reasoned argument will require ranges of data sources and ranges of study types.

Although one needs to be cautious about the interpretation of the data, equally, one cannot allow the perfect to become the enemy of the good. There are no perfect ways to analyse a data set such as this one, and yet the question of the role of country-level governance in health systems performance is an important one, that has received little attention. In the absence of any analysis, no matter how imperfect, there can be no debate; and this analysis at least places the issues "on the table" and invites the collection of better data. Governance is significantly associated with health outcomes. If the association is causal, and one might anticipate that it is [14], then it would be unwise to ignore that possibility because of data quality issues. The unanticipated relationship between finance and health equity certainly warrants further consideration. Ways forward include (unsatisfactorily) waiting for the development of better time-series data, and (positively) the development of focussed case-studies within a handful of countries.

## Conclusion

There are a range of indicators, largely under-utilised, that could provide important points for intervention in population health. Recent data from large scale studies such as the Commission on Social Determinants of Health provided important and alternative ways of conceptualising the factors that should be within the purview of the health sector. Analyses such as the one presented here provide a powerful argument for inter-sectoral approaches to population health improvement outside the health or medical care sector, notwithstanding weaknesses in the data. The indication that governance matters for health system performance is of importance for policy making as it could point to structural interventions that have greater impact on health outcomes than individually targeted interventions.

The assessment of healthcare systems alone as a proxy for health systems performance in countries where broader determinants such as governance and infrastructure remain inadequate is restrictive both in making progress and assessing improvements. More research is needed to support our finding, research that uses different type of data such as time series data or even case studies applying qualitative measures.

## Competing interests

The authors declare that they have no competing interests.

## Authors' contributions

AEO designed the study, conducted the analysis and wrote the first draft of a report. DDR supported the analysis and created a preliminary draft based on AEO's report. SP participated in the design of the analysis and PA contributed to the discussion and write up of the paper. All authors read and approved the final manuscript.

## Pre-publication history

The pre-publication history for this paper can be accessed here:

http://www.biomedcentral.com/1471-2458/11/237/prepub

## References

[B1] World Health Organization The World Health Report 2000 Health Systems: Improving Performance 2000

[B2] Lakhani A Coles J Eayres D Spence C Rachet B Creative use of existing clinical and health outcomes data to assess NHS performance in England: Part 1--performance indicators closely linked to clinical care BMJ 2005 330 7505 1426 1432 10.1136/bmj.330.7505.1426 15961815 PMC558380

[B3] Westert G van den Berg M Kooman X Verkleij H Dutch Health Care Performance Report 2008 2008

[B4] Schoen C Davis K How SKH Schoenbaum SC U.S. Health System Performance: A National Scorecard Health Aff 2006 25 w457 w475 10.1377/hlthaff.25.w457 16987933

[B5] OECD OECD Health Project (2001-2004) 2004 http://www.oecd.org/document/28/0,3343,en_2649_33929_2536540_1_1_1_37407,00.htm Accessed 05/22, 2009

[B6] Arah OA Westert GP Hurst J Klazinga NS A conceptual framework for the OECD Health Care Quality Indicators Project Int J Qual Health Care 2006 18 suppl_1 5 13 10.1093/intqhc/mzl024 16954510

[B7] Nolte E McKee M Population health in Europe: how much is attributable to health care? World Hospitals and Health Services 2004 40 3 12 14 40, 42 15566272

[B8] McKee M Suhrcke M Nolte E Lessof S Figueras J Duran A Health systems, health, and wealth: a European perspective The Lancet 2009 373 9660 349 351 10.1016/S0140-6736(09)60098-2 19167573

[B9] Hanson K Gilson L Goodman C Mills A Smith R Feachem R Is Private Health Care the Answer to the Health Problems of the World's Poor? PLoS Medicine 2008 5 11 e233 10.1371/journal.pmed.0050233 19067483 PMC2586358

[B10] Armstrong Schellenberg J Mrisho M Manzi F Shirima K Mbuya C Mushi A Health and survival of young children in southern Tanzania BMC Public Health 2008 8 1 194 10.1186/1471-2458-8-194 18522737 PMC2442074

[B11] Kristiansson C Gotuzzo E Rodriguez H Bartoloni A Strohmeyer M Tomson G Access to health care in relation to socioeconomic status in the Amazonian area of Peru International Journal for Equity in Health 2009 8 1 11 10.1186/1475-9276-8-11 19368731 PMC2680861

[B12] Travis P Bennett S Haines A Pang T Bhutta Z Hyder AA Overcoming health-systems constraints to achieve the Millennium Development Goals The Lancet 2004 364 9437 900 906 10.1016/S0140-6736(04)16987-0 15351199

[B13] Murray CJ Frenk JA A framework for assessing the performance of health systems Bulletin of the World Health Organization 2000 78 6 717 731 10916909 PMC2560787

[B14] Commission on Social Determinants of Health editor Closing the gap in a generation 2008 1 Geneva: World Health Organization

[B15] Carrin G Xu K Evans DB Exploring the features of universal coverage Bulletin of the World Health Organization 2008 86 11 818 10.2471/BLT.08.060137 19030678 PMC2649567

[B16] World Bank World Development report 1993: Investing in Health 1993 http://files.dcp2.org/pdf/WorldDevelopmentReport1993.pdf Accessed 03/25, 2010

[B17] Singh-Manoux A Dugravot A Smith GD Subramanyam M Subramanian SV Adult education and child mortality in India: the influence of caste, household wealth, and urbanization Epidemiology 2008 19 2 294 301 10.1097/EDE.0b013e3181632c75 18300716 PMC3056118

[B18] McAlister C Baskett TF Female education and maternal mortality: a worldwide survey Journal of obstetrics and gynaecology Canada 2006 28 11 983 990 17169224 10.1016/S1701-2163(16)32294-0

[B19] Ako AA Nkeng GE Takem GEE Water quality and occurrence of water-borne diseases in the Douala 4th District, Cameroon Water science and technology 2009 59 12 2321 2329 10.2166/wst.2009.268 19542637

[B20] Kaufmann D Kraay A Mastruzzi M Governance Matters VIII: Aggregate and Individual Governance Indicators, 1996-2008 2009 http://papers.ssrn.com/sol3/papers.cfm?abstract_id=1424591 Accessed 6/1, 2009

[B21] Kaufmann D Kraay A Zoido P Governance Matters 1999 http://papers.ssrn.com/sol3/papers.cfm?abstract_id=188568 Accessed 11/8, 2010

[B22] Reidpath D Allotey P Structure, (governance) and health: an unsolicited response BMC International Health and Human Rights 2006 6 1 12 10.1186/1472-698X-6-12 16978401 PMC1584251

[B23] United Nations Development Programme Statistics of the Human Development Report 2008 http://hdr.undp.org/en/statistics/Accessed 02/04, 2009

[B24] World Health Organization World Health Statistics 2008 2008 http://www.who.int/whosis/whostat/EN_WHS08_Table1_Mort.pdf Accessed 02/06, 2009

[B25] Mo Ibrahim Foundation The Ibrahim Index 2010 http://www.moibrahimfoundation.org/en/section/the-ibrahim-index Accessed 01/25, 2011

[B26] Blaxter M The Health of Children: A Review of Research on the Place of Health in Cycles of Disadvantage 1981

[B27] Reidpath D Allotey P Infant mortality rate as an indicator of population health J Epidemiol Community Health 2003 57 5 344 6 10.1136/jech.57.5.344 12700217 PMC1732453

[B28] Reidpath DD Morel CM Mecaskey JW Allotey P The millennium development goals fail poor children: the case for equity-adjusted measures PLoS Medicine 2009 6 4 e1000062 10.1371/journal.pmed.1000062 19399155 PMC2667271

[B29] Gwatkin DR Rutstein S Johnson K Suliman E Wagstaff A Amouzou A Socio-Economic Differences in Health, Nutrition, and Population Within Developing Countries 2007 Washington DC: World Bank 18293634

[B30] Braveman P Gruskin S Defining equity in health Journal of Epidemiology and Community Health 2003 57 4 254 258 10.1136/jech.57.4.254 12646539 PMC1732430

[B31] Reidpath DD Allotey P Measuring global health inequity International Journal for Equity in Health 2007 6 16 10.1186/1475-9276-6-16 17971215 PMC2147004

[B32] Graham J Amos P Plumptre T Principles for Good Governance in the 21st Century 2003 http://www.iog.ca/publications/policybrief15.pdf Accessed 20/05, 2009

[B33] Saisana M Annoni P Nardo M A robust model to measure governance in African countries. Report No. EUR23773 EN 2990 2009 European Commission Joint Research Centre. Ispra Varese, Italy

[B34] Gelman A Hill J Data Analysis Using Regression and Multilevel/Hierarchial Models 2007 Cambridge, England: Cambridge University Press

